# [^177^Lu]Lu-DOTATATE Population Pharmacokinetics and Dosimetry Modeling for Adolescent and Adult Patients with Somatostatin Receptor–Positive Gastroenteropancreatic Neuroendocrine Tumors

**DOI:** 10.2967/jnumed.125.270202

**Published:** 2026-06

**Authors:** Meemansa Sood, Larissa Lachi Silva, Yu-Yun Ho, Lars Blumenstein, Azzeddine Cherfi, Lingfei Xu, Fariba Khanshan

**Affiliations:** 1 Novartis Pharma AG, Basel, Switzerland;; 2 Novartis Pharmaceuticals Corporation, East Hanover, New Jersey;; 3 Novartis Institutes for BioMedical Research, Basel, Switzerland; and; 4 Novartis Pharmaceuticals Corporation, Cambridge, Massachusetts

**Keywords:** radioligand therapy, RLT, radiopharmaceutical therapy, RPT, ^177^Lu-DOTATATE, adolescent dosage extrapolation, population pharmacokinetics modeling, exposure–dosimetry modeling

## Abstract

There is an unmet need for treatment options for adolescents with malignant advanced gastroenteropancreatic neuroendocrine tumors or pheochromocytomas and paragangliomas. Whether radiopharmaceutical therapy dosing strategies for adults are suitable for adolescents is unknown. This study compared the plasma exposure and cumulative absorbed radiation dose of [^177^Lu]Lu-DOTATATE (^177^Lu-DOTATATE) between adults and adolescents treated with 7.4 GBq for 4 cycles every 8 wk to assess the probability of adolescent doses exceeding predefined safety thresholds for kidney and bone marrow dosimetry. **Methods:** Population pharmacokinetics (popPK) and exposure–dosimetry models for ^177^Lu-DOTATATE, developed for adults, and simulations based on the exposure–dosimetry model, were used to determine sample size for the NETTER-P trial, assuming that the same dosage is administered to adolescents as to adults. Subsequently, a popPK model on adolescents and an exposure–dosimetry model on pooled adult and adolescent data were developed, and the results were compared with those from adult models. **Results:** PopPK models for adults and adolescents showed no significant covariate effects. Applying an adult dosimetry model to an adolescent population predicted median kidney and bone marrow dosimetry values below predefined safety threshold levels of 29 Gy or less and 2 Gy or less, respectively. PopPK modeling confirmed comparable ^177^Lu-DOTATATE exposure, and kidney and bone marrow dosimetry values were predicted to be largely below threshold levels. The observed proportions and median predicted probabilities of exceeding thresholds after 4 cycles were approximately 20% for kidney or significantly below 20% for bone marrow, which was considered an acceptable safety limit. Age and weight did not show a clinically significant impact on ^177^Lu-DOTATATE exposure or kidney and bone marrow dosimetry values, confirming that flat dosing at adult dosage is appropriate for adolescents. **Conclusion:** ^177^Lu-DOTATATE with 7.4 GBq of activity, administered over 4 cycles 8 wk apart, is a well-tolerated therapeutic dosing regimen for adolescent patients with gastroenteropancreatic neuroendocrine tumors or pheochromocytomas and paragangliomas.

## Visual Abstract

**Figure:**
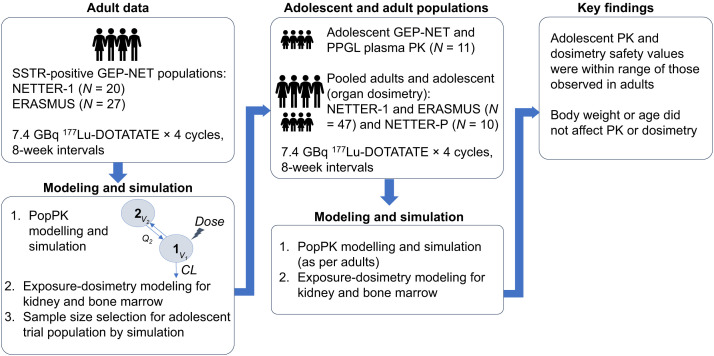

Gastroenteropancreatic neuroendocrine tumors (GEP-NETs) are a heterogeneous type of neuroendocrine neoplasm, with a globally rising incidence rate (1). Although less common in children, most GEP-NETs lead to poor health-related quality of life (2,3) and are often diagnosed at later stages (4–6), with lower survival rates (7). Currently, there is a lack of evidence regarding the optimal treatment strategies for GEP-NETs, particularly tumors of advanced grade (8).

[^177^Lu]Lu-DOTATATE (^177^Lu-DOTATATE) is a β-particle–emitting radiopharmaceutical therapy with high affinity for somatostatin receptor (SSTR) type 2 (9), expressed by most GEP-NETs (10). ^177^Lu-DOTATATE was approved in the European Union in 2017 for the treatment of adults with unresectable or metastatic, progressive, well-differentiated grade 1 or grade 2 SSTR-positive GEP-NETs (11). It was also approved by the Food and Drug Administration in 2018 for the treatment of adults with SSTR-positive GEP-NETs including foregut, midgut, and hindgut tumors (12). Approval was based on findings from the phase 3 NETTER-1 trial (13) and the supportive phase 1/2 ERASMUS multicenter trial (14). In adults with advanced or metastatic SSTR-positive GEP-NETs, ^177^Lu-DOTATATE has been proven efficacious and well tolerated, with a positive impact on progression-free survival (13–17). The approved administered radioactivity of ^177^Lu-DOTATATE in adults is 7.4 GBq every 8 wk for 4 cycles (referred to hereafter as dosage), for a total cumulative administered activity of 29.6 GBq. Based on data from external beam radiation threshold (EBRT) studies, this dosage is considered safe for the kidneys and bone marrow (18–20), the primary organs at risk of potential toxicity.

In adolescents, treatment options are limited, especially for advanced disease. Case studies suggest that ^177^Lu-DOTATATE is an appropriate treatment for this population (21,22), but previous studies have been conducted predominantly in adults. The NETTER-P trial is a multicenter, open-label, single-arm phase 2 study designed to evaluate the safety and dosimetry of intravenously delivered ^177^Lu-DOTATATE in adolescents aged 12 to 18 y with SSTR-positive GEP-NETs or SSTR-positive pheochromocytomas and paragangliomas (PPGLs) (23). PPGLs were included because these show features similar to those of GEP-NETs. Secondary outcomes include comparison of pharmacokinetics and dosimetry assessments between adolescents with GEP-NETs and PPGLs as a pooled cohort, and adult patients, using population pharmacokinetics (popPK) and exposure–dosimetry models.

To ensure that the adult dosage is suitable for adolescents, an efficacy extrapolation from adults to adolescents was investigated. To allow for efficacy extrapolation, the relationship between activity, radiolabeled plasma concentrations, dosimetry, and demographic factors was investigated for adults and adolescents. The analysis used data from 2 ^177^Lu-DOTATATE studies in adults: NETTER-1 (NCT01578239) (13) and ERASMUS (14). Extrapolation was primarily focused on comparing either the plasma exposure of ^177^Lu-DOTATATE or the cumulative activity already established in adults with adolescents and evaluating the effect on kidney and bone marrow EBRT thresholds. ^177^Lu-DOTATATE dosimetry limits were explored on the basis of data from EBRT and radiopharmaceutical therapy studies with various radioisotopes, which recommend exposure thresholds for different organs (e.g., a conservative bone marrow threshold limit of 2 Gy) (24,25). For kidneys, published data vary in terms of specific threshold limits for ^177^Lu-DOTATATE and radiopharmaceutical therapies (19,26–28); therefore, 2 proposed thresholds were explored: 23 Gy (26) and 29 Gy (28).

This work tested whether adolescents with GEP-NETs or PPGLs can be treated with the same dosage of ^177^Lu-DOTATATE as that used for adults with GEP-NETs without significant risk of exceeding the predefined threshold limits for kidney and bone marrow dosimetry.

## MATERIALS AND METHODS

### Patient Populations

This study adhered to the Declaration of Helsinki, the International Conference on Harmonisation E6 Good Clinical Practice, and applicable regulations and was approved by institutional review boards or independent ethics committees at each center. Written informed consent or assent was obtained from all participants or their legal guardians before study participation. Data were collected by investigators and analyzed by the sponsor, Advanced Accelerator Applications, a Novartis company.

#### Adult Population

Data used were from NETTER-1 (NCT01578239) (13) and ERASMUS (14). PopPK analysis included 20 patients with advanced midgut neuroendocrine tumors from NETTER-1. Dosimetry analyses included 47 patients with GEP-NETs (27 ERASMUS and 20 NETTER-1).

#### Adolescent Population

Data from 11 patients from NETTER-P (NCT04711135) (23)—4 patients with SSTR-positive GEP-NETs and 7 with SSTR-positive PPGLs—were used in this analysis. Additional information is available in supplemental materials (available at http://jnm.snmjournals.org) (29,30) and in Gaze et al. (31). Data cutoff was March 12, 2024.

### Full Efficacy Extrapolation

The full efficacy extrapolation approach used for adolescents (Supplemental Fig. 1) was based on assumptions made regarding similar disease characteristics, mechanism of action, pharmacokinetics, biodistribution, and organ dosimetry between the adult and adolescent populations per the Food and Drug Administration guidelines (32). This approach was based on findings from the NETTER-1 study, where radiolabeled plasma pharmacokinetics was not influenced by patient characteristics, and unpublished findings from NETTER-1 and ERASMUS, where activity and renal function (as determined by creatinine clearance [CrCL]) were explanatory covariates of kidney and bone marrow dosimetry. Weight-adjusted dosing should not apply to ^177^Lu-DOTATATE in adolescents, since no effects of weight, age, or body surface area were observed on pharmacokinetics or biodistribution in adults.

### Determination of Adolescent Trial Size Through Modeling and Simulation of Adult Data

#### PopPK for Adults

The adult (NETTER-1) popPK dataset was created from actual mass dose, radioactivity–blood pharmacokinetics (converted to mass), demographics, and laboratory information at baseline. A popPK model was developed, using a 2-compartment, 0-order input with first-order elimination (Supplemental Eqs. 1 and 2).

Using MonolixSuite 2021R2 software, individual pharmacokinetics parameters were estimated via empiric Bayes estimates, corresponding to the most likely value in their conditional distributions. Covariate selection including baseline characteristics (e.g., age, weight, CrCL, and kidney size) on ^177^Lu-DOTATATE plasma pharmacokinetics was performed. The final NETTER-1 model was used to derive individual exposure metrics based on actual administration records using Simulx 2021R2. PopPK-predicted exposure metrics were not calculated for ERASMUS patients; therefore, activity was used as a surrogate of exposure for subsequent exposure–dosimetry analysis.

#### Exposure–Dosimetry Modeling for Adults

Empiric models were used to describe the relationship between ^177^Lu-DOTATATE kidney and bone marrow dosimetry and covariates. As only 1 dosimetry value per subject was available, nonlinear regression was performed for each organ separately. Covariates were selected after exploration of their relationship with the kidney and bone marrow dosimetry. Model building was undertaken based on reduction in Bayesian information criterion and consideration of the diagnostic plots, particularly visual predictive check (VPC). Equations used to estimate model parameters for the adult population are as follows:

$$\mathrm{kidney}\;\mathrm{dosimetry}=A_{\mathrm{pop}}\times \left[{\left[\frac{\mathrm{COV}1}{\mathrm{median}\;\mathrm{COV}1}\right]}^{B_{\mathrm{pop}}}\times {\left[\frac{\mathrm{COV}2}{\mathrm{median}\;\mathrm{COV}2}\right]}^{C_{\mathrm{pop}}}\right],$$ Eq. 1

$$\mathrm{bone}\;\mathrm{marrow}\;\mathrm{dosimetry}=D_{\mathrm{pop}}\times \left[{\left[\frac{\mathrm{COV}3}{\mathrm{median}\;\mathrm{COV}3}\right]}^{E_{\mathrm{pop}}}\times {\left[\frac{\mathrm{COV}4}{\mathrm{median}\;\mathrm{COV}4}\right]}^{F_{\mathrm{pop}}}\right],$$ Eq. 2

where *A*_pop_, *B*_pop_, *C*_pop_, *D*_pop_, *E*_pop_, and *F*_pop_ are fixed effects and COV1, COV2, COV3, and COV4 refer to covariates in a generic sense.

To establish kidney and bone marrow activity–response patterns as a function of selected covariates, simulations explored activities (1–8 GBq) across a range of possible covariate values, for example, CrCL (35–180 mL/min), creating 240 scenarios. For each activity–covariate pair, 500 virtual subjects were simulated (supplemental materials).

### Sample Size Determination

The NETTER-P trial size was determined through a simulation to assess the probability that the median kidney dosimetry of a clinical trial population may exceed a limit of 23 or 29 Gy. A random sample of 2,500 and 5,000 adolescents was simulated independently from the CrCL distribution defined by Piepsz et al. (33), at an activity of 7.4 GBq for 4 cycles in adults. These samples were further divided into 500 trials comprising 5 or 10 virtual subjects. The median kidney dosimetry was calculated for each of these 500 trials using the dosimetry model developed on the adult population. The probability of the median kidney dosimetry exceeding 23 and 29 Gy was calculated. Based on these probability distributions, it was determined that 8 would be a sufficient sample size to be enrolled in NETTER-P.

### Statistical Analysis: Modeling and Simulation on Adolescent Data

#### PopPK for Adolescents

The base popPK model structure was the same as that for adult patients. Population and individual pharmacokinetics parameters were estimated in MonolixSuite 2021R1 using the same approach as that described for the adult popPK model. Covariate selection was performed in NETTER-P using 2 methods (supplemental materials). The final NETTER-P model was used to derive individual exposure metrics based on actual administration records for subsequent exposure–dosimetry analyses.

#### Exposure–Dosimetry Analyses for Pooled Adults and Adolescents

As the adolescent population was relatively small, exposure–dosimetry analyses were based on a pooled dataset of adults and adolescents (*n* = 57; 47 adults from NETTER-1 and ERASMUS studies, and 10 adolescents from the NETTER-P study), and potential correlations between exposure metrics, demographics, and other baseline features with kidney and bone marrow dosimetry were evaluated in the pooled dataset (Eqs. 1 and 2).

## RESULTS

Demographic data are shown in Table 1.

**TABLE 1.** ** **Demographic Data of Adult (*n* = 47) and Adolescent (*n* = 11) Populations

Parameter	Adults	Adolescents
Age (y)	56 (29–83)	15 (13–17)
Body weight (kg)	75 (48–145)	55 (39.5–71)
BSA (m^2^)	1.9 (1.48–2.68)	1.58 (1.3–1.85)
CrCL (mL/min)	98.82 (46.97–189.77)	122.1 (86–160)
Kidney mass (g)	339 (201–575)*	273.7 (169.3–346.7)
Sex		
Male	24 (51.1%)	5 (45.5%)
Female	23 (48.9%)	6 (54.5%)
Indication		
GEP-NET	47 (100%)	4 (36.4%)
PPGL		7 (63.6%)
Study		
NETTER-1	20 (42.55%)	
ERASMUS	27 (57.45%)	
NETTER- P		11 (100%)

* Data available only for 20 NETTER-1 patients.

BSA = body surface area.

Qualitative data are number and percentage, and continuous data are median and range.

### PopPK Modeling and Simulation for Adults

**FIGURE 1.**
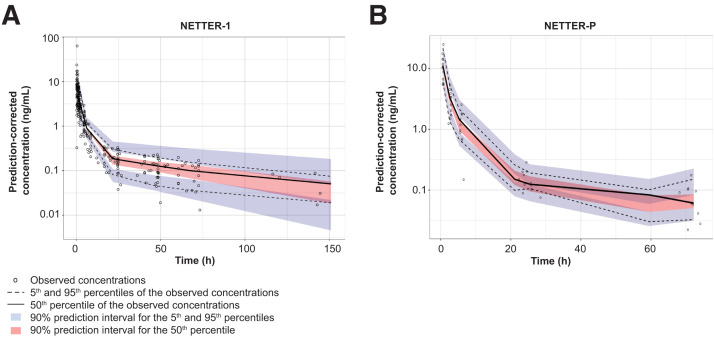
PopPK prediction-corrected VPC for plasma concentration comparing adult (A) vs. adolescent (B) populations.

The final popPK model showed no covariate effect. All parameters were well estimated (Table 2) for adults, and clearance and its interindividual variability were estimated at high precision, at a relative SE (RSE) of 9.81% and 18.29%, respectively. The population clearance and volume of distribution of the central compartment (*V*_c_) were estimated to be 4.95 L/h and 21.59 L, respectively, with interindividual variability on clearance estimated to be 0.41 and that on *V*_c_ to be 0.52. The constant residual error of the model was estimated at a low value of 0.4. None of the baseline characteristics (e.g., age, weight, body surface area) were significant covariates on pharmacokinetics parameters (clearance or *V*_c_). The adult population showed evidence of deviation from high observed values (underprediction) (Supplemental Fig. 2A); however, this was the case only for 1 patient, likely due to rapid drug infusion (<0.5 h). The prediction-corrected VPC of the final model for NETTER-1 showed good predictability, with median observations lying within the 90% prediction interval (Fig. 1A).

**TABLE 2.** Adult PopPK Parameter Estimates

**Parameter**	**NETTER-1 estimate**
CL (L/h)	4.95 (9.81)
*V*_c_ (L)	21.59 (12.70)
*Q* (L/h)	4.78 (6.69)
*V*_p_ (L)	202.15 (10.12)
IIV on CL	0.41 (18.29)
IIV on *V*_c_	0.52 (19.37)
Cor CL ∼ *V*_c_	0.70 (20.79)
Constant residual error	0.40 (4.85)

CL = clearance from central compartment; *Q* = intercompartmental clearance; *V*_p_ = volume of distribution of peripheral compartment; IIV = interindividual variability; Cor CL ∼ *V*_c_ = correlation between CL and *V*_c_.

Values in parentheses are %RSE. %RSE is defined as SE × 100%.

### Exposure–Dosimetry Modeling and Simulation for Adults

The adult popPK-predicted area under the curve from time 0 to infinity showed no correlation with kidney dosimetry values (*R* = −0.027, *P* = 0.91) but had a positive correlation with bone marrow dosimetry values (*R* = 0.58, *P* = 0.0075) (Supplemental Figs. 3A and 3B). However, exposure data for area under the curve from time 0 to infinity were available only from NETTER-1. To include ERASMUS subjects in the dosimetry analysis, activity was used as a surrogate for exposure.

Final dosimetry model equations with the parameter estimates (Table 3) for the adult population were as follows:

$$\mathrm{kidney}\;\mathrm{dosimetry}=4.3\times \left[{\left[\frac{\mathrm{activity}\;(\mathrm{GBq})}{7.4\;(\mathrm{GBq})}\right]}^{0.66}\times {\left[\frac{\mathrm{CrCL}\;(\mathrm{mL}/\min)}{99\;(\mathrm{mL}/\min)}\right]}^{\text{−}0.552}\right],$$ Eq. 3

$$\mathrm{bone}\;\mathrm{marrow}\;\mathrm{dosimetry}=0.246\times \left[{\left[\frac{\mathrm{activity}\;\left(\mathrm{GBq}\right)}{7.4\;(\mathrm{GBq})}\right]}^{0.597}\times {\left[\frac{\mathrm{CrCL}\;(\mathrm{mL}/\min)}{99\;(\mathrm{mL}/\min)}\right]}^{\text{−}1.11}\right].$$ Eq. 4

**TABLE 3.** Final Dosimetry Model Parameters Estimated for Adult Population (*n* = 47)

Parameter	Estimate with study effect
*A*_pop_, kidney baseline	4.3 (8.37%)
*B*_pop_, activity effect on kidney	0.66 (23%)
*C*_pop_, CrCL effect on kidney	−0.552 (37.3%)
*D*_pop_, bone marrow baseline	0.246 (11%)
*E*_pop_, activity effect on bone marrow	0.597 (35.6%)
*F*_pop_, CrCL effect on bone marrow	−1.11 (24.4%)
*b*_1_	0.515 (12.8%)
*b*_2_	0.675 (14.3%)

*b*_1_ = proportional residual error for kidney model (additive variance); *b*_2_ = proportional residual error for bone marrow model (additive variance).

Values in parentheses are %RSE. %RSE is defined as SE × 100%.

**FIGURE 2.**
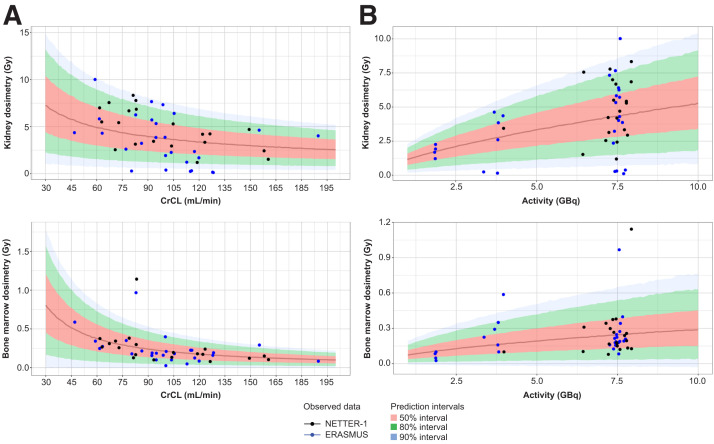
Adult kidney and bone marrow VPCs in terms of organ dosimetry vs. CrCL (A) and activity (B), showing NETTER-1 and ERASMUS study populations.

The VPCs based on 500 simulations (Fig. 2) showed that the dosimetry models adequately described the observed dosimetry data for the kidney and bone marrow based on CrCL and dosage for the adult population. The adequacy of the models was also demonstrated by goodness-of-fit plots (Supplemental Fig. 4).

### Determination of Adolescent Trial Size

**FIGURE 3.**
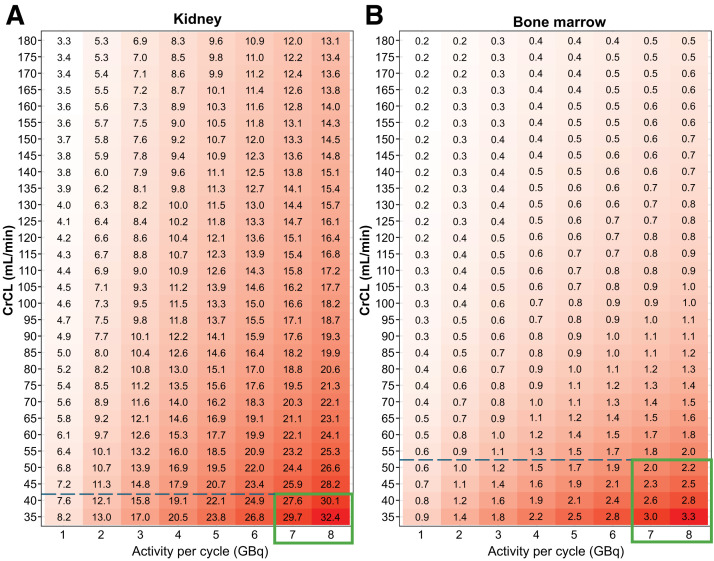
Median dosimetry estimates from 500 virtual subjects in 240 scenarios of varying activities per cycle and CrCL values for 4 treatment cycles as function of CrCL value and activity per cycle in kidney (A) and bone marrow (B).

Figure 3 shows the median dosimetry from 500 virtual subjects of each of the 240 scenarios of activity paired with CrCL. For a CrCL greater than 55 mL/min, predicted median dosimetry values were lower than for the predefined kidney (≤29 Gy) and bone marrow (≤2 Gy) thresholds at the adult dosage. Median dosimetry estimates from 500 virtual subjects for 4 treatment cycles as a function of CrCL are shown in Supplemental Table 1, and the predicted probability of the kidney and bone marrow dosimetry exceeding 29 and 2 Gy, respectively, is shown in Supplemental Table 2.

**FIGURE 4.**
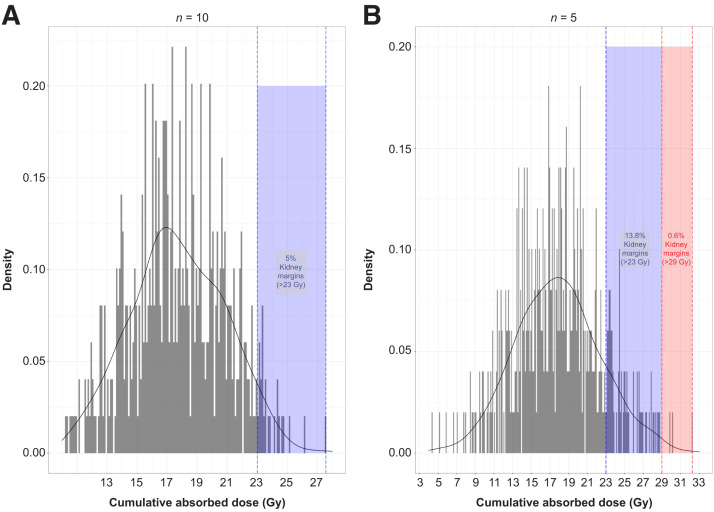
Sample size determination for NETTER-P population based on distribution of median kidney dosimetry from 500 trials of 10 (A) and 5 (B) randomly selected adolescents.

The probability of the median cumulative absorbed dose in the kidneys exceeding 23 and 29 Gy was calculated, and the distribution of median trial dosimetry values were displayed against these thresholds (Fig. 4). The median kidney dosimetry values were calculated for 500 simulated trials with an *n* of 5 and 10. Predicted probabilities of the median trial kidney dosimetry exceeding the 23 Gy threshold were 5% (*n* = 10) and 13.8% (*n* = 5), respectively, and those for exceeding the 29 Gy threshold were 0% (*n* = 10) and 0.6% (*n* = 5). In both cases, the probabilities were well below the predefined 20% limit.

### Modeling and Simulation for Adolescents

#### PopPK Modeling and Simulation for Adolescents

In NETTER-P, because of the small sample size, a horseshoe prior approach was used to further explore the covariate effect on clearance. No statistically significant covariates on clearance were found (Supplemental Fig. 5).

Like NETTER-1, a model without a covariate effect was selected as the final popPK model for NETTER-P. All parameters in the final model were estimated with reasonable precision. The clearance for an adolescent patient was estimated at 5.92 L/h (%RSE, 5.43); *V*_c_ was 17.86 L (%RSE, 7.22), and interindividual variability on clearance was 0.14 (%RSE, 30.72) (Table 4). The constant residual error was low, estimated at 0.25. The NETTER-P popPK model-predicted plasma concentrations described the observed pharmacokinetics concentrations well (Supplemental Fig. 2B). The prediction-corrected VPCs of the final models showed good model predictability for both NETTER-1 (Fig. 1A) and NETTER-P (Fig. 1B).

**TABLE 4.** ** **Adolescent PopPK Parameter Estimates

Parameter	NETTER-P estimate
CL (L/h)	5.92 (5.43)
*V*_c_ (L)	17.86 (7.22)
*Q* (L/h)	2.63 (8.89)
*V*_p_ (L)	99.42 (15.04)
IIV on CL	0.14 (30.72)
Constant residual error	0.25 (10.62)

CL = clearance from central compartment; *Q* = intercompartmental clearance; *V*_p_ = volume of distribution of peripheral compartment; IIV = interindividual variability.

Values in parentheses are %RSE. %RSE is defined as SE × 100%.

**FIGURE 5.**
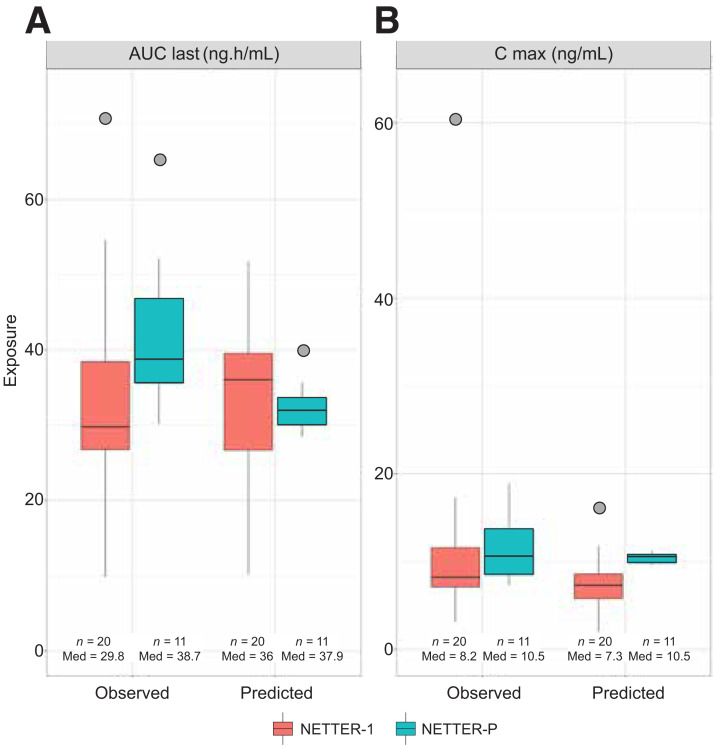
Pharmacokinetics exposure (observed and predicted) in adult and adolescent populations. Area under curve from dosing to time of last measured concentration (AUC last), area under concentration–time curve from time 0 to time of last measured concentration. C max = maximum concentration; Med = median.

Pharmacokinetics metrics, including area under the curve from dosing to the time of the last measured concentration, maximum plasma concentration, and the time to reach maximum plasma concentration were calculated and compared between 20 adults from NETTER-1 and 11 adolescents from NETTER-P, as previously reported (31). We confirmed similar pharmacokinetics exposure metrics, including variability, between adults and adolescents (Fig. 5).

#### Exposure–Dosimetry Modeling and Simulation for Pooled Adolescent and Adult Data

A dosimetry model similar to the adult model (Table 2) was fitted using activity (exposure metric) and CrCL as covariates for the kidney and bone marrow based on pooled adult and adolescent data (Table 5). Different models were tested. The study effect, which can be assumed as adult versus adolescent population, was used instead of age. For kidney dosimetry, the final model showed a different relationship between CrCL and dosimetry depending on population (Eq. 5). Although adding study as a covariate for the kidney dosimetry model improved the model fit, this result should be interpreted with caution because of the small sample size of the adolescent population (*n* = 10). For kidney dosimetry, the predicted cumulative absorbed dose based on the final model for adults and adolescents for cycle 1 was calculated as 4.37 and 5.39 Gy, respectively, below the 29 Gy threshold for 4 cycles. For bone marrow dosimetry, no improvement in parameter estimates was observed when additional covariates were added. The final dosimetry model equations for pooled data with the parameter estimates (Table 5) are detailed below:

$$\mathrm{kidney}\;\mathrm{dosimetry}=4.37\times [{\left[\frac{\mathrm{activity}\;(\mathrm{GBq})}{7.4}\right]}^{0.65}\times {\left[\frac{\mathrm{CrCL}}{99}\right]}^{\left(\text{−}0.55[+1.58\;\text{if}\;\text{study}\;\text{is}\;\text{NETTER-P}]\right)}],$$ Eq. 5

$$\mathrm{bone}\;\mathrm{marrow}\;\mathrm{dosimetry}=0.24\times \left[{\left[\frac{\mathrm{activity}\;(\mathrm{GBq})}{7.4}\right]}^{0.52}\times {\left[\frac{\mathrm{CrCL}}{99}\right]}^{\text{−}1.18}\right].$$ Eq. 6

**TABLE 5.** ** **Final Dosimetry Model Parameters Estimated for Pooled Adults and Adolescents (*n* = 57)

Parameter	Estimate with study effect
*A*_pop_, kidney baseline	4.37 (7.22)
*B*_pop_, activity effect on kidney	0.65 (22.0)
*C*_pop_, CrCL effect on kidney	−0.55 (35.1)
beta_C_STUDY_NETTER_P, CrCL effect on kidney dosimetry based on adult or adolescent populations	1.58 (37.5)
*D*_pop_, bone marrow baseline	0.24 (8.90)
*E*_pop_, activity effect on bone marrow	0.52 (37.8)
*F*_pop_, CrCL effect on bone marrow	−1.18 (19.7)
*b*_1_	0.48 (11.3)
*b*_2_	0.63 (12.5)

*b*_1_ = proportional error for kidney model; *b*_2_ = proportional error for bone marrow model.

Values in parentheses are %RSE. %RSE is defined as SE × 100%.

**FIGURE 6.**
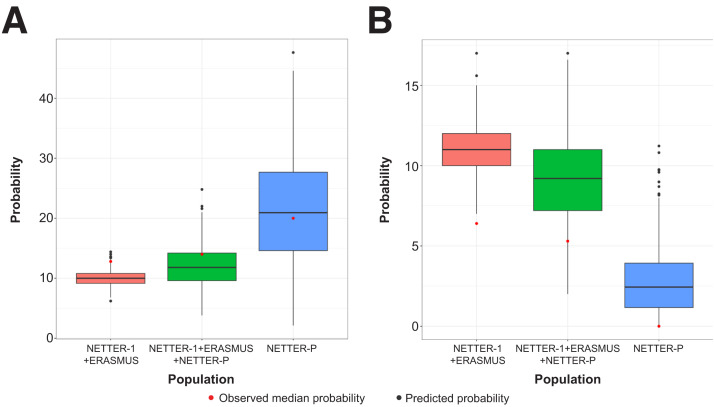
Predicted probability of exceeding kidney (A) or bone marrow (B) dosimetry thresholds in adult and adolescent populations. Predicted probabilities were summarized by median (5th and 95th percentiles) across 500 simulated trials, each with 500 virtual subjects.

**FIGURE 7.**
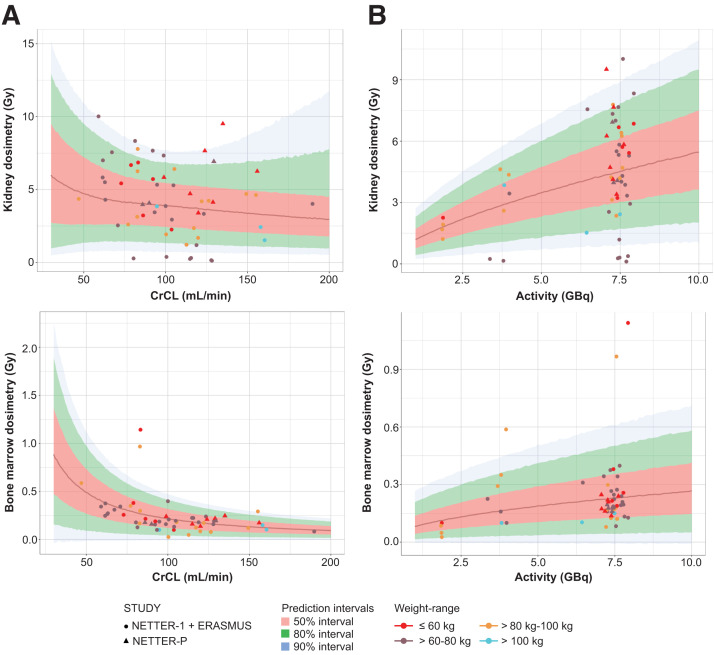
Flat dosing confirmation using pooled adult and adolescent populations: organ dosimetry vs. CrCL (A) and activity (B).

Predicted probabilities of exceeding the defined thresholds for kidney and bone marrow dosimetry were calculated from the final dosimetry model based on pooled data (Fig. 6) using the same data as those from the NETTER-P publication (31). The results showed predicted dosimetry values to be largely under the threshold levels. The median observed and predicted probabilities of kidney dosimetry values exceeding 29 Gy and bone marrow dosimetry exceeding 2 Gy thresholds after an adult dosage were approximately 20% for kidney and significantly below 20% for bone marrow, respectively. Data from both populations fell within a similar range and were also similar among different weight groups, confirming the appropriateness of flat dosing (Fig. 7).

## DISCUSSION

Clinical development of ^177^Lu-DOTATATE initially targeted adults with inoperable, progressive, SSTR-positive GEP-NETs. Here, adolescent dosage selection was based on confirming similar exposure and dosimetry relationships between adults and adolescents using the same dosage. PopPK-predicted exposure metrics were similar for both adults and adolescents, confirming similarity between both populations regarding ^177^Lu-DOTATATE exposure. Bone marrow exposure–dosimetry relationships were also comparable between adults and adolescents. For kidney, “study” was added as a covariate to address differing CrCL effects, which showed an opposite trend in adolescents (dosimetry increased with CrCL). Interpretation is limited by small sample size and absence of renal impairment. Predicted probabilities of bone marrow dosimetry values exceeding 2 Gy were slightly lower for adolescents. Predicted probabilities of kidney dosimetry values exceeding 29 Gy were higher in adolescents (∼20%) than in adults (10%–13%), aligned with the observed proportions from the pooled studies. Neither ^177^Lu-DOTATATE exposure nor organ dosimetry was impacted by age or body weight in a clinically relevant manner, confirming that flat dosing of 7.4 GBq of ^177^Lu-DOTATATE administered every 8 wk over 4 cycles in adults is appropriate for adolescents aged 12 to 18 y.

Since CrCL significantly impacts kidney dosimetry, a decrease in renal function should result in increased dosimetry estimates. Nevertheless, dosimetry simulations predicted that median kidney and bone marrow radiation exposures would remain within safe limits if CrCL remained above 55 mL/min. However, for kidney dosimetry, CrCL can be as low as 40 mL/min. ^177^Lu-DOTATATE is well tolerated in both adult and adolescent patients, and the associated nephrotoxicity is mild (34,35), even in patients with chronic kidney disease (36). However, studies on the effects of whole-body radiotherapy in pediatrics are limited and mainly concern patients with Wilms tumor, who are usually younger than those with GEP-NETs at diagnosis and often undergo unilateral or bilateral nephrectomy (37,38). As dosimetry estimates were below EBRT thresholds, and the observed probabilities of exceeding 29 Gy for kidneys and 2 Gy for bone marrow after 4 treatment cycles with 7.4 GBq in both adult and adolescent populations did not significantly exceed the 20% acceptable safety limit, the adult recommendations can be considered when treating renally impaired adolescent patients. Thus, whereas a careful benefit–risk assessment should be performed before treatment of adolescents with baseline renal impairment, dosage modifications are not required in adolescents with renal dysfunction based on CrCL values.

Although GEP-NETs and PPGLs occur relatively rarely in children, metastatic disease can be difficult to manage, and there is a great unmet medical need. Therefore, ^177^Lu-DOTATATE offers an efficient treatment option for patients with unresectable disease. These findings, in addition to those from the primary analysis of NETTER-P (31), led to Food and Drug Administration approval of ^177^Lu-DOTATATE in April 2024 as the first treatment for patients aged 12 y or older with SSTR-positive GEP-NETs (39). Small sample size is a limitation of this work.

## CONCLUSION

This work compared adolescent and adult patient populations, demonstrating similar ^177^Lu-DOTATATE plasma exposure and acceptable safety profiles not exceeding predefined EBRT thresholds, based on exposure–dosimetry analyses in the kidneys and bone marrow in both populations. These results confirm that an adult dosage of ^177^Lu-DOTATATE with 7.4 GBq administered every 8 wk for 4 cycles is appropriate for adolescents.

## DISCLOSURE

This study was funded by Novartis AG, Basel, Switzerland. Meemansa Sood, Larissa Lachi Silva, Yu-Yun Ho, Lars Blumenstein, Azzeddine Cherfi, and Lingfei Xu are Novartis employees; Fariba Khanshan was a Novartis employee at the time of participation in this study. Meemansa Sood, Azzeddine Cherfi, Yo-Yun Ho, and Larissa Lachi Silva are Novartis shareholders. No other potential conflict of interest relevant to this article was reported.

## Notes

KEY POINTS **QUESTION:** Is the ^177^Lu-DOTATATE dosage established for adults suitable for adolescents aged 12 to 18 y? **PERTINENT FINDINGS:** Plasma exposure and exposure–dosimetry characteristics in dose-limiting organs were similar for adults and adolescents, demonstrating that an adult dosage of 7.5 GBq of ^177^Lu-DOTATATE administered every 8 wk for 4 cycles to deliver a total cumulative administered activity of 29.6 GBq can be safely administered to adolescents. **IMPLICATIONS FOR PATIENT CARE:** ^177^Lu-DOTATATE is a well-tolerated treatment option for adolescents with SSTR-positive GEP-NETs or PPGLs.
